# Supplementary material for: Transmission of SARS-CoV-2 in domestic cats imposes a narrow bottleneck
Source: bioRxiv. 2021 Jan 4:2020.11.16.384917. Originally published 2020 Nov 17. Preprint. [Version 2] doi: 10.1101/2020.11.16.384917 (PMC7685321; doi:10.1101/2020.11.16.384917)
Supplement: Supplement 1 [file media-1.pdf]

Supplementary Figures

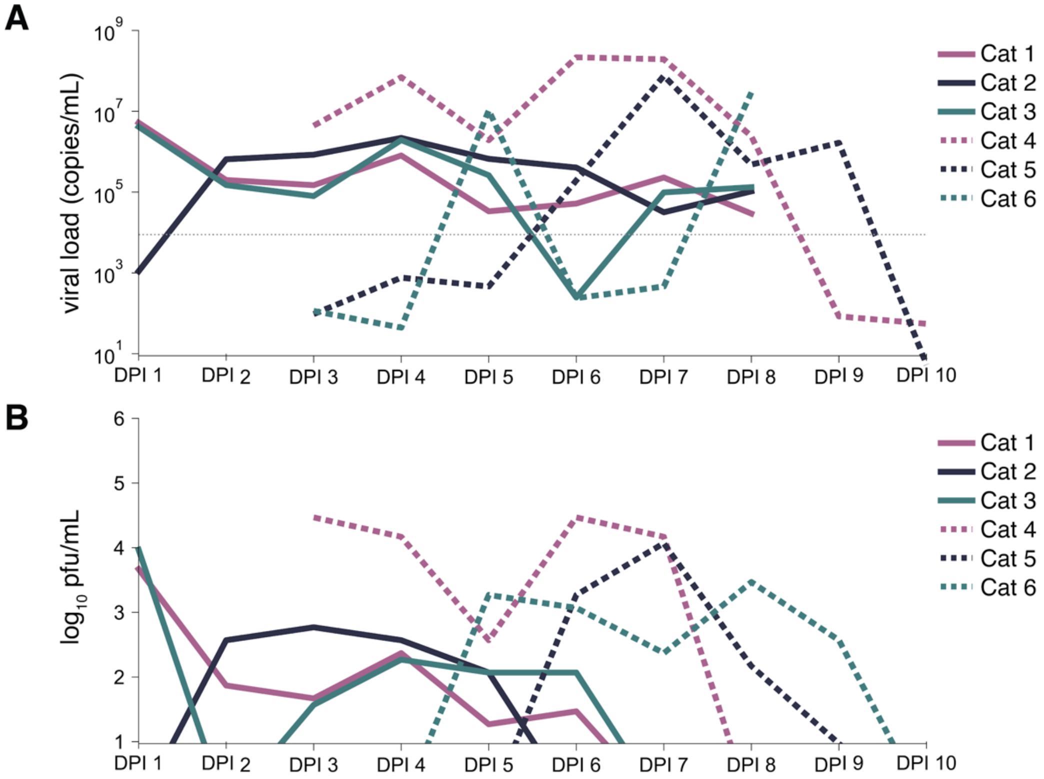

Supplementary Figure 1. **Viral loads and viral titers over time**. A) Viral RNA burden over time for each cat. Index cats are represented by a solid line and contact cats are represented by a dashed line. Transmission pairs are denoted by color. The grey, horizontal dotted line represents when less than ~100 copies/μL are input into the reverse transcription reaction. B) Infectious viral titer over time. Index cats are represented by a solid line and contact cats are represented by a dashed line. Transmission pairs are denoted by color.

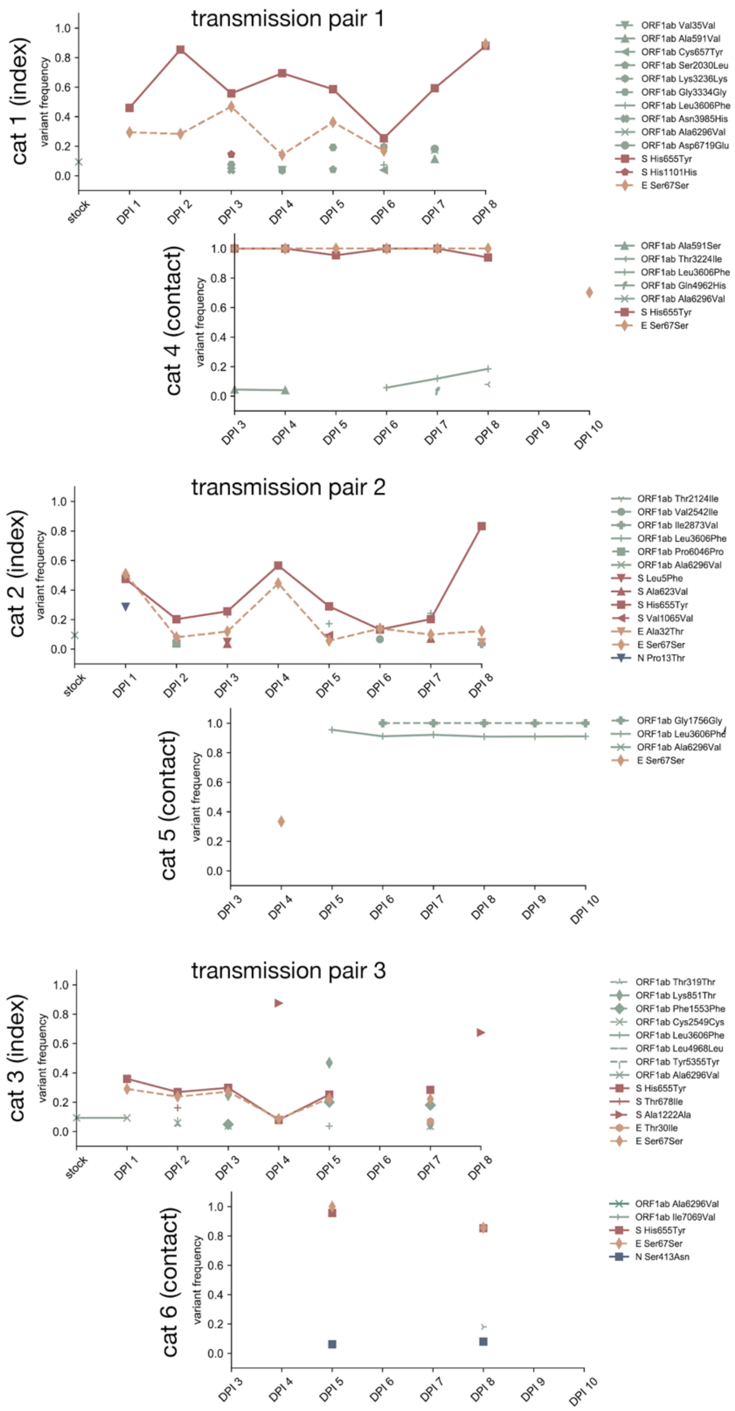

Supplementary Figure 2. Longitudinal frequency of iSNVs detected in all cats and at all timepoints. Each variant is colored based on gene location. Nonsynonymous variants are plotted with solid lines and synonymous variants are plotted with dashed lines. Days with viral loads too low to yield high quality sequences are shown by the gaps in data (i.e. cat 3 day 6 and cat 4 day 9).

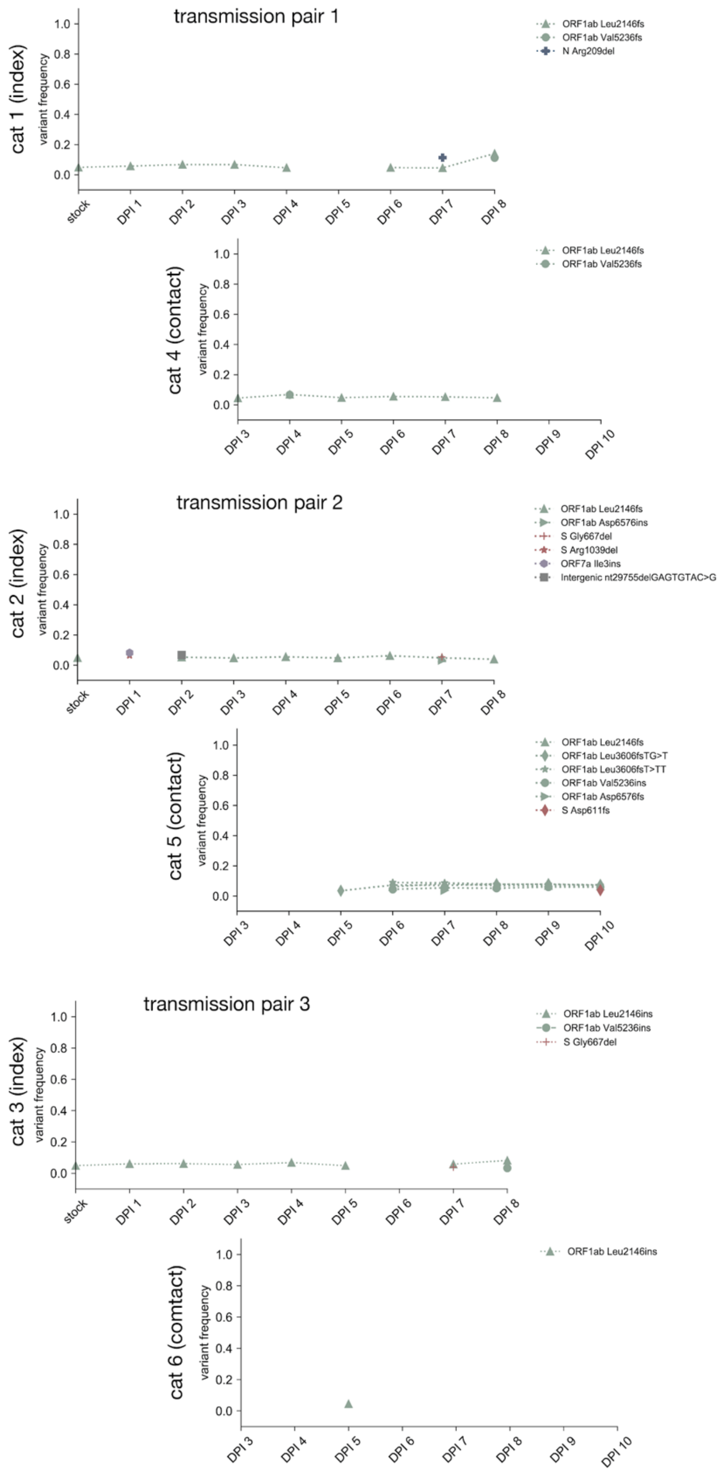

Supplementary Figure 3. Longitudinal frequency of indels detected in all cats and at all timepoints. Each indel is colored based on gene location. Days with viral loads too low to yield high quality sequences are shown by the gaps in data (i.e. cat 3 day 6 and cat 4 day 9).

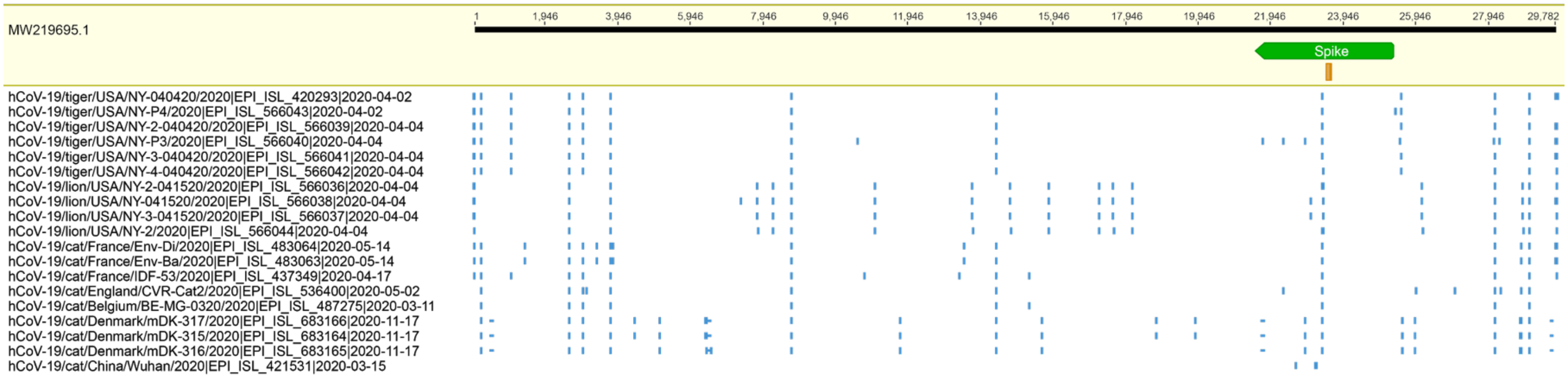

Supplementary Figure 4. Sequence alignment of all tiger, lion, and domestic cat sequences available in GISAID as of December 2020. Sequences were aligned against MW219695.1, the inoculum virus used in these experiments. Consensus-level differences are highlighted with a blue vertical marker. Indels are noted with a horizontal vertical marker. The spike open reading frame is annotated with a green marker and site amino acid 655 in Spike is highlighted with the orange box. None of these sequences contain a consensus mutation at residue 655 in Spike.

A

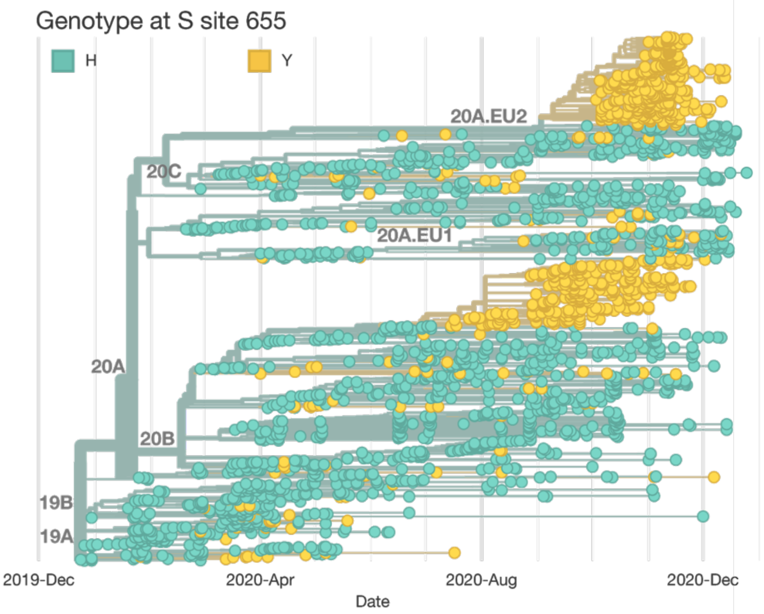

B

Country	Date of first detected sequence with H655Y	Number of sequences	Date of most recent detected sequence with H655Y
United Kingdom	2020-03-13	943	2020-12-13
Denmark	2020-04-13	44	2020-11-30
USA	2020-03-01	42	2020-11-12
South Africa	2020-06-11	8	2020-10-9
India	2020-05-11	6	2020-9-2
China	2020-01-26	3	2020-3-13
Netherlands	2020-05-07	3	2020-12-7
Australia	2020-07-31	3	2020-8-12
Israel	2020-03-24	2	2020-4-5
United Arab Emirates	2020-05-15	2	2020-5-18
France	2020-05-26	2	2020-8-14
Rwanda	2020-10-17	2	2020-10-22
Taiwan	2020-03-19	1	2020-3-19
Portugal	2020-04-02	1	2020-4-2
Belize	2020-04-09	1	2020-4-9
Romania	2020-04-09	1	2020-4-9
Turkey	2020-04-26	1	2020-4-26
Brazil	2020-06-30	1	2020-6-30
Bangladesh	2020-07-12	1	2020-7-12
Czech Republic	2020-11-04	1	2020-11-4
Russia	2020-11-16	1	2020-11-16
Italy	2020-12-02	1	2020-12-2

C Geographic dispersion of SARS-CoV-2 viruses with Spike H655Y variant

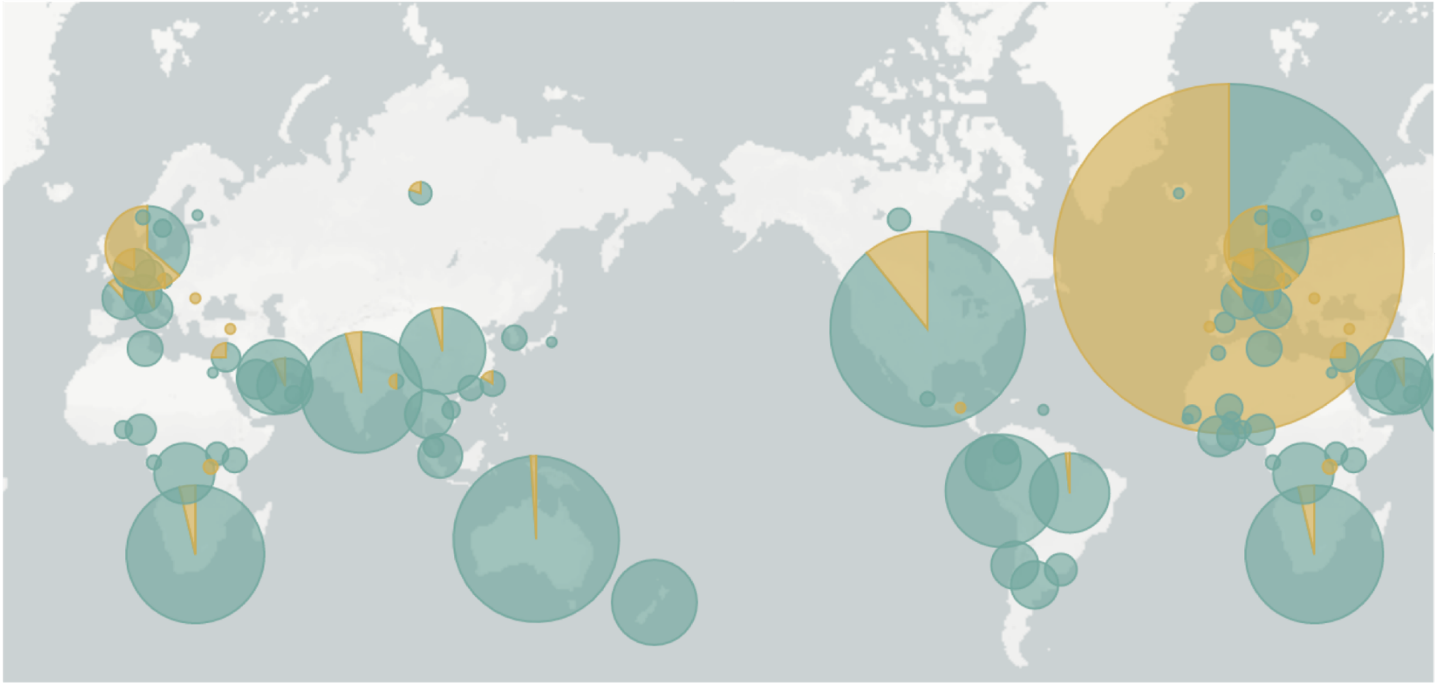

Supplementary Figure 5. Geographic dispersion of Spike H655Y variant. A) A time-resolved phylogeny focused on viruses that contain Spike H655Y. Viruses that contain histidine (H) at Spike 655 are colored in teal. Viruses with tyrosine (Y) at Spike 655 are colored in yellow. B) Counts of SARS-CoV-2 viruses that contain Spike H655Y, broken down by country. C) Map highlighting the number viruses from each country. The size of the circle represents the number of sequences from the appropriate country contained in the phylogeny.

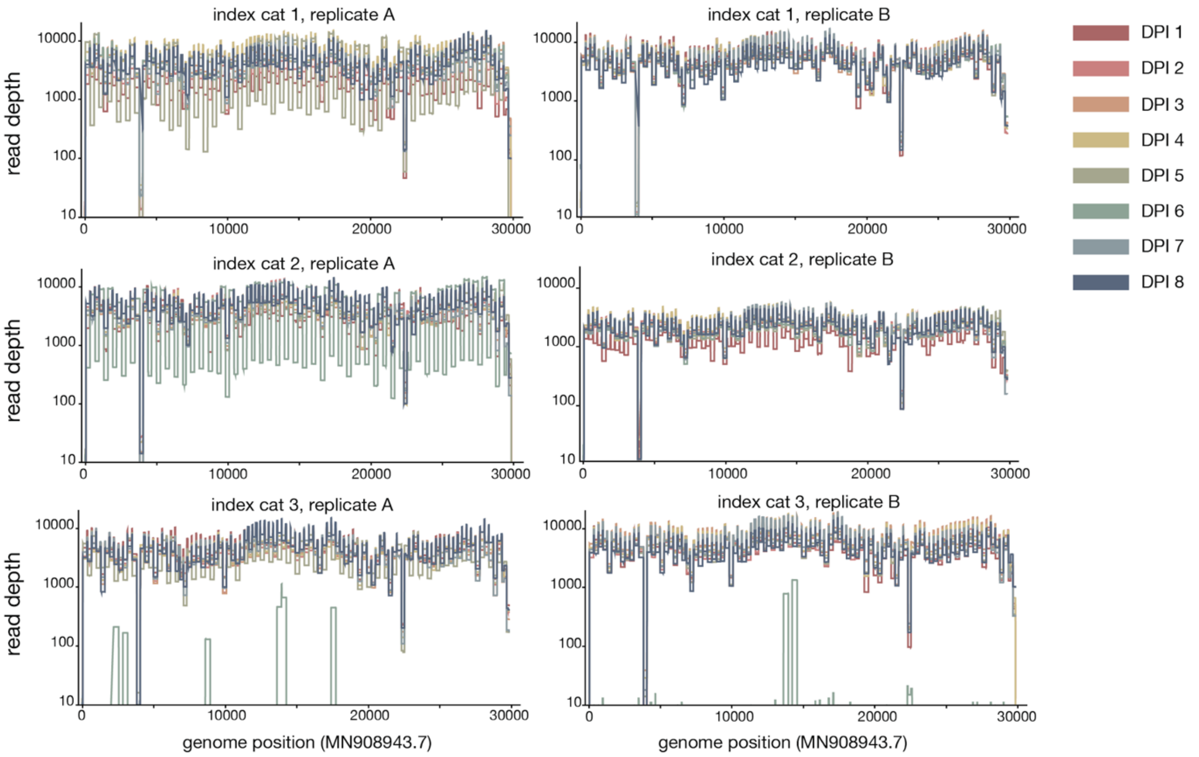

Supplementary Figure 6. Read depth across the SARS-CoV-2 genome in index cats. Each day is represented by a different color. Replicate A is shown in the left column and replicate B is shown in the right column.

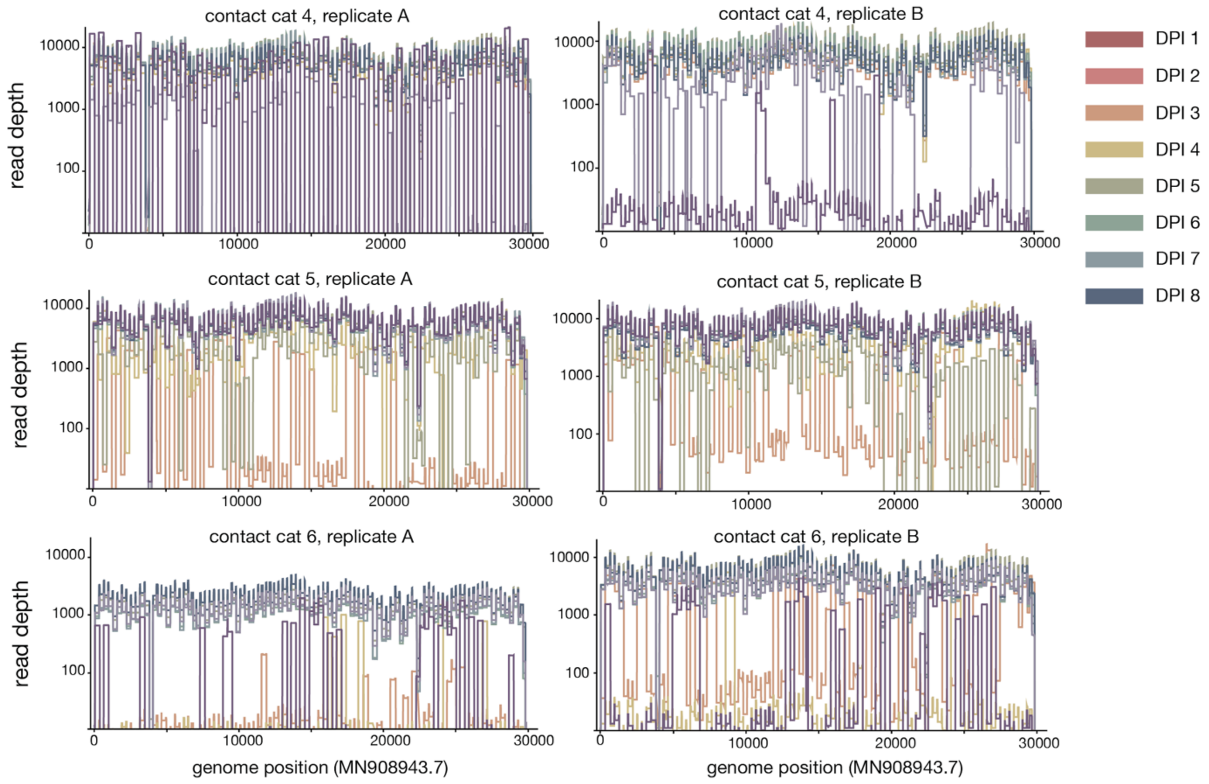

Supplementary Figure 7. Read depth across the SARS-CoV-2 genome in contact cats. Each day is represented by a different color. Replicate A is shown in the left column and replicate B is shown in the right column.

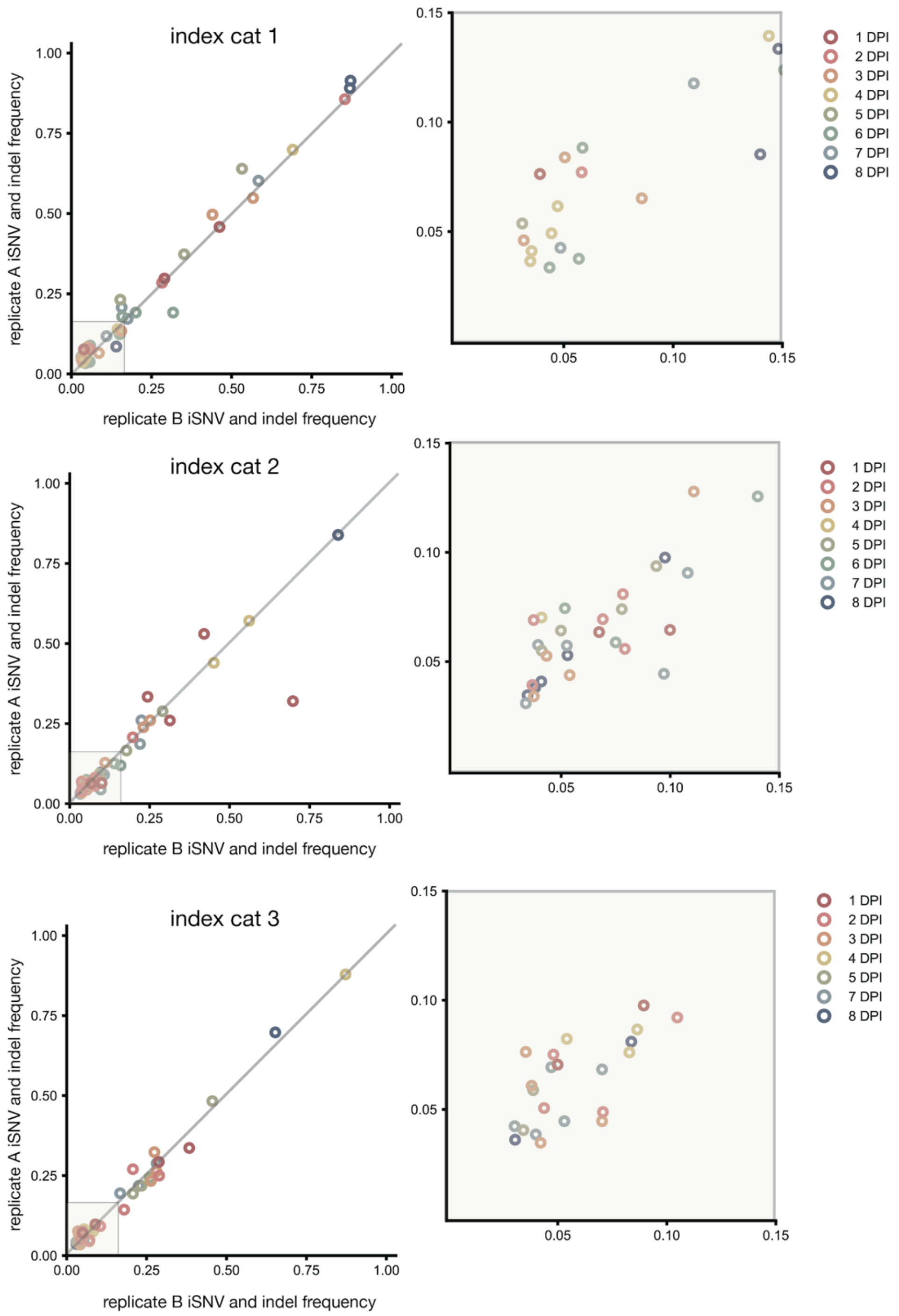

Supplementary Figure 8. Intersection variants found across technical replicates in index cats. The frequency of each variant per replicate is shown here. The diagonal line represents the 1:1 intersection of replicate variants. The subplot to the right of each primary plot is a zoomed-in view of the low-frequency variants (3-15%). Each timepoint is denoted by a different color.

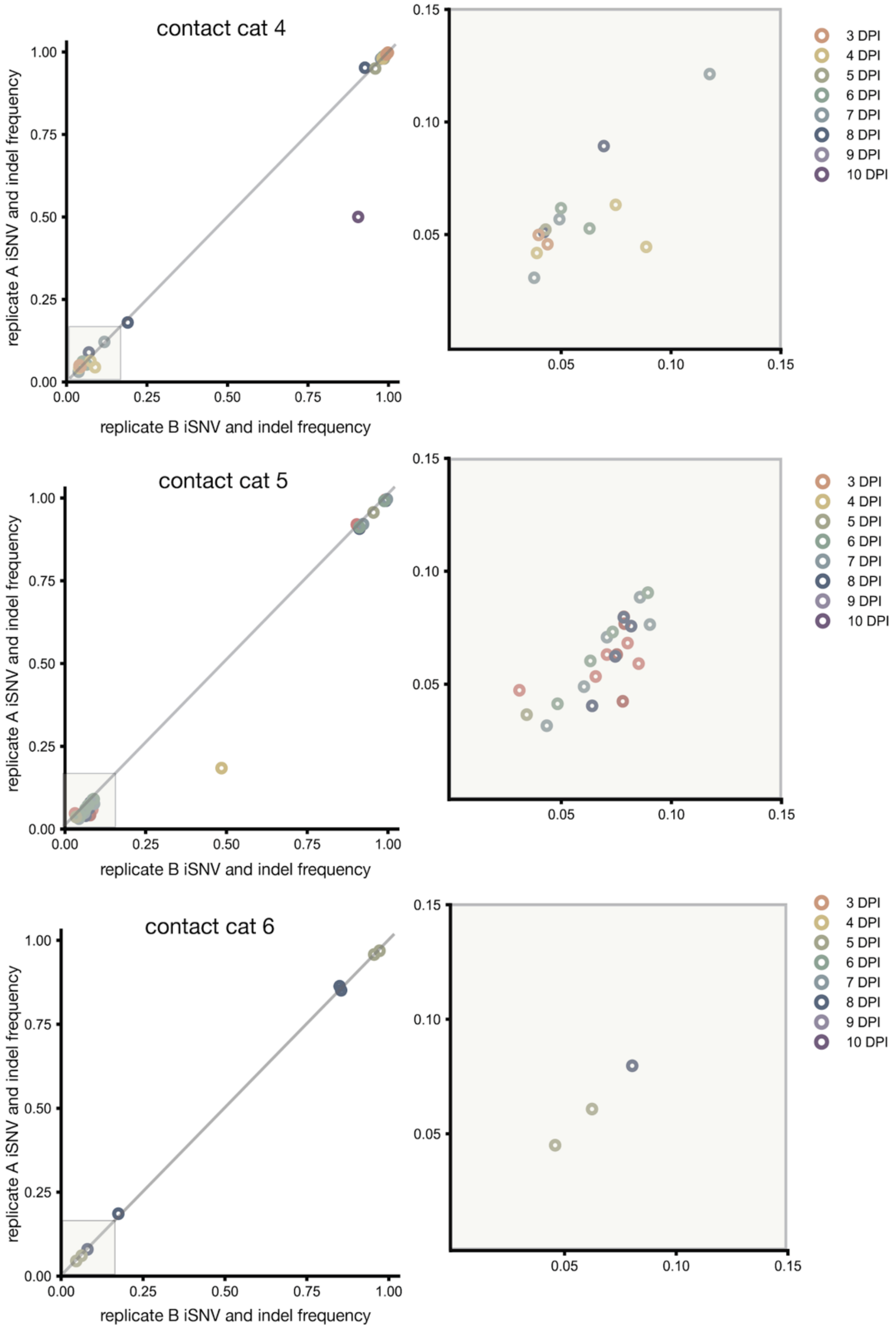

Supplementary Figure 9. Intersection variants found across technical replicates in contact cats. The frequency of each variant per replicate is shown here. The diagonal line represents the 1:1 intersection of replicate variants. The subplot to the right of each primary plot is a zoomed-in view of the low-frequency variants (3-15%). Each timepoint is denoted by a different color.

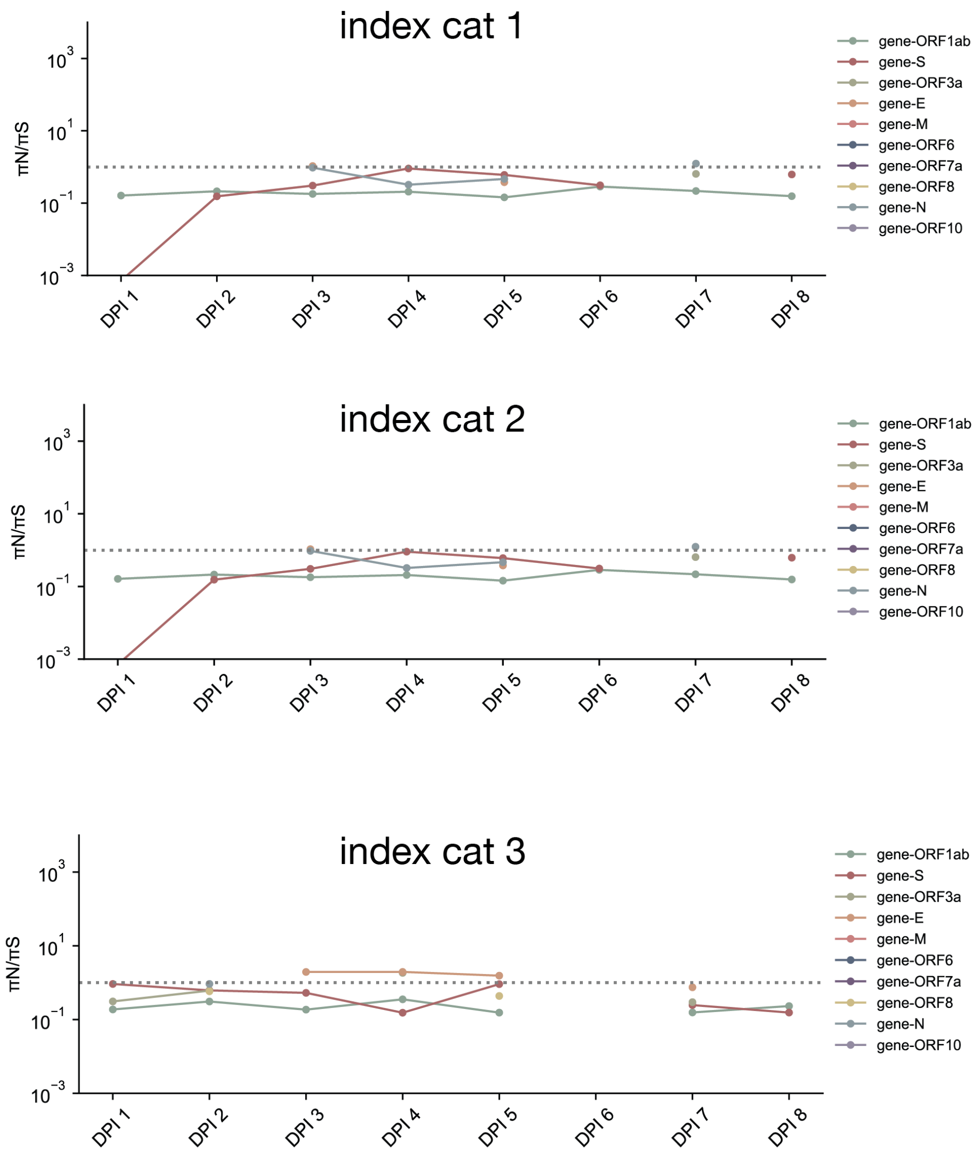

Supplementary Figure 10. Longitudinal pairwise nonsynonymous nucleotide diversity divided by pairwise synonymous nucleotide diversity in index cats. Line color denotes gene. The horizontal dotted gray line is plotted at y = 1 or when πN ~ πS.

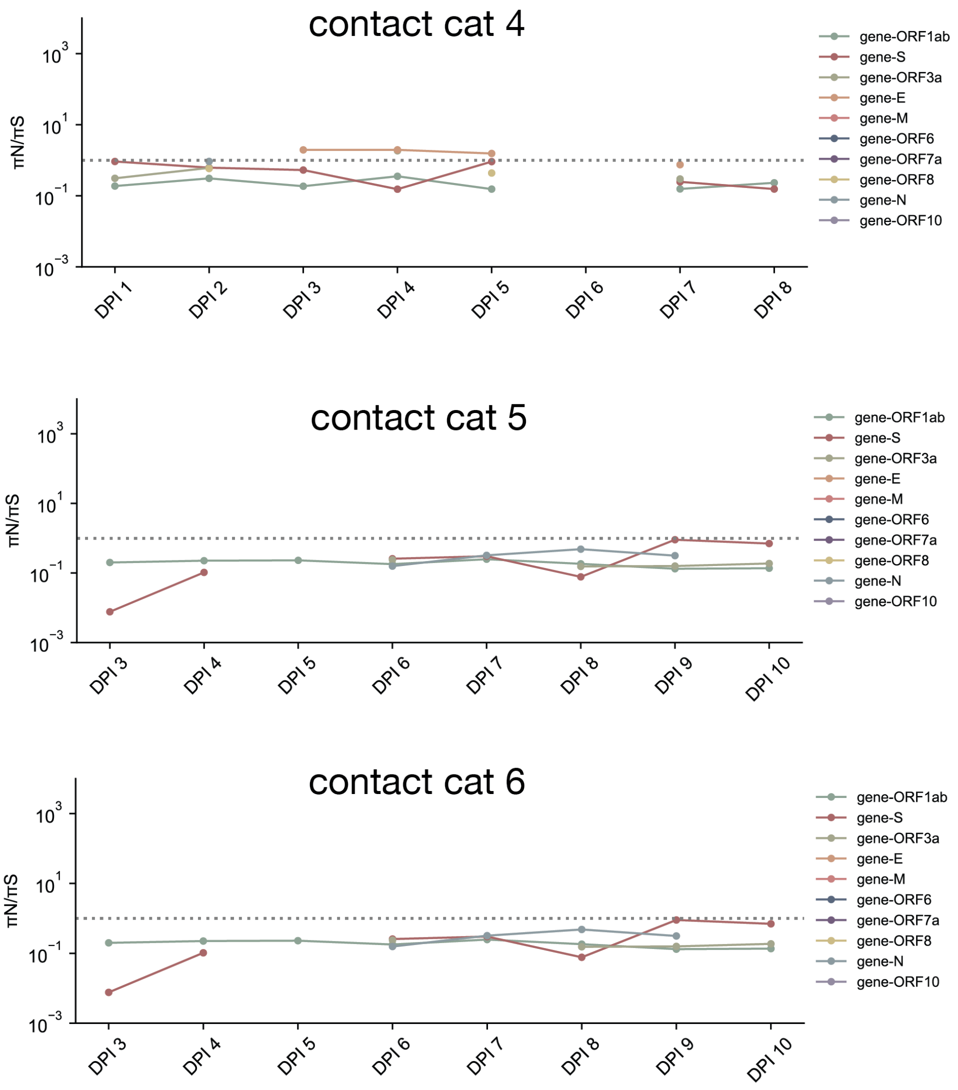

Supplementary Figure 11. Longitudinal pairwise nonsynonymous nucleotide diversity divided by pairwise synonymous nucleotide diversity in contact cats. Line color denotes gene. The horizontal dotted gray line is plotted at $y = 1$ or when πN ~ πS.

Supplementary Tables

	mean πS	std πS	mean πN	std πN	πN/πS	statistic, p-value
index cat 1						
**ORF1ab**	0.015670	0.005609	0.003019	0.001124	0.195265	(statistic=6.23, pvalue=2.11e-05)
**S**	0.001564	0.000600	0.000644	0.000467	0.413995	(statistic=3.34, pvalue=0.005)
**ORF3a**	0.005367	0.000001	0.005878	0.002267	0.641899	(statistic=-0.303, pvalue=0.772)
**E**	0.011707	0.010139	0.011930	0.005719	0.719601	(statistic=-0.0456, pvalue=0.965)
**M**			0.002138	0.000059		(statistic=nan, pvalue=nan)
**ORF6**			0.007395			(statistic=nan, pvalue=nan)
**ORF7a**	0.011992	0.000487				(statistic=nan, pvalue=nan)
**ORF8**	0.031186	0.012756				(statistic=nan, pvalue=nan)
**N**	0.005292	0.002941	0.003036	0.001403	0.744626	(statistic=1.410, pvalue=0.196)
**ORF10**						(statistic=nan, pvalue=nan)
index cat 2						
**ORF1ab**	0.025570	0.007229	0.005423	0.001061	0.219071	(statistic=7.799, pvalue=1.837e-06)
**S**	0.004651	0.002567	0.001476	0.000556	0.457442	(statistic=3.419, pvalue=0.0042)
**ORF3a**	0.008660	0.003052	0.003867	0.001630	0.535879	(statistic=3.549, pvalue=0.0053)
**E**	0.008410	0.009696	0.015842	0.010396	436.372255	(statistic=-1.191, pvalue=0.261)
**M**			0.002479	0.000841		(statistic=nan, pvalue=nan)
**ORF6**			0.007468			(statistic=nan, pvalue=nan)
**ORF7a**	0.011872					(statistic=nan, pvalue=nan)
**ORF8**	0.030343	0.012115	0.005673	0.002620	0.224566	(statistic=2.733, pvalue=0.0292)
**N**	0.006988	0.004962	0.001398	0.000535	0.346156	(statistic=2.240, pvalue=0.066)
**ORF10**						(statistic=nan, pvalue=nan)
index cat 3						
**ORF1ab**	0.022195	0.005336	0.004752	0.000970	0.225357	(statistic=8.509, pvalue=1.988e-06)
**S**	0.003619	0.001697	0.001584	0.001032	0.505292	(statistic=2.711, pvalue=0.0189)
**ORF3a**	0.005516	0.000037	0.004351	0.003509	0.764893	(statistic=0.649, pvalue=0.533)
**E**	0.016174	0.000532	0.023985	0.007123	1.553581	(statistic=-2.145, pvalue=0.0642)
**M**	0.006492		0.002078	0.000007		(statistic=nan, pvalue=nan)
**ORF6**						(statistic=nan, pvalue=nan)
**ORF7a**	0.012294	0.000305				(statistic=nan, pvalue=nan)
**ORF8**	0.020154	0.006891	0.008740	0.002221	0.509847	(statistic=2.708, pvalue=0.0352)
**N**	0.005320	0.001974	0.003336		0.925883	(statistic=nan, pvalue=nan)
**ORF10**						(statistic=nan, pvalue=nan)

Supplementary Table 1. Nonsynonymous and synonymous nucleotide diversity estimates in index cats.

	Cat 1	Cat 2	Cat 3	Cat 4	Cat 5	Cat 6
**π DPI 1**	0.000246	0.000290	0.000433			
**π DPI 2**	0.000314	0.000705	0.000546			
**π DPI 3**	0.000458	0.000557	0.000781	0.000712	0.000153	
**π DPI 4**	0.000577	0.000650	0.000568	0.000796	0.000206	0.000037
**π DPI 5**	0.000489	0.000513	0.000540	0.001007	0.000149	0.000576
**π DPI 6**	0.000430	0.000720		0.000917	0.000854	0.000156
**π DPI 7**	0.000365	0.000541	0.000683	0.000721	0.000876	0.000025
**π DPI 8**	0.000214	0.000591	0.000458	0.000879	0.000872	0.000720
**π DPI 9**				0.000125	0.000965	0.000070
**π DPI 10**				0.000371	0.000932	0.000000
**mean π**	0.000387	0.000571	0.000573	0.000691	0.000626	0.000226
**std π**	0.000117	0.000128	0.000114	0.000279	0.000355	0.000273

Supplementary Table 2. Genome-wide pairwise nucleotide diversity estimates in index and contact cats

name	pool	sequence	length	%gc	tm (use 65)
nCoV-2019_1_LEFT	nCoV-2019_1	ACCAACCAACTTTCGATCTCTTGT	24	41.67	60.69
nCoV-2019_1_RIGHT	nCoV-2019_1	CATCTTTAAGATGTTGACGTGCCTC	25	44	60.45
nCoV-2019_2_LEFT	nCoV-2019_2	CTGTTTTACAGGTTCGCGACGT	22	50	61.67
nCoV-2019_2_RIGHT	nCoV-2019_2	TAAGGATCAGTGCCAAGCTCGT	22	50	61.74
nCoV-2019_3_LEFT	nCoV-2019_1	CGGTAATAAAGGAGCTGGTGGC	22	54.55	61.32
nCoV-2019_3_RIGHT	nCoV-2019_1	AAGGTGTCTGCAATTCATAGCTCT	24	41.67	60.32
nCoV-2019_4_LEFT	nCoV-2019_2	GGTGTATACTGCTGCCGTGAAC	22	54.55	61.56
nCoV-2019_4_RIGHT	nCoV-2019_2	CACAAGTAGTGGCACCTTCTTTAGT	25	44	60.97
nCoV-2019_5_LEFT	nCoV-2019_1	TGGTGAAACTTCATGGCAGACG	22	50	61.39
nCoV-2019_5_RIGHT	nCoV-2019_1	ATTGATGTTGACTTTCTCTTTTTGGAGT	28	32.14	60.17
nCoV-2019_6_LEFT	nCoV-2019_2	GGTGTTGTTGGAGAAGGTTCCG	22	54.55	61.64
nCoV-2019_6_RIGHT	nCoV-2019_2	TAGCGGCCTTCTGTAAAACACG	22	50	61.18
nCoV-2019_7_LEFT	nCoV-2019_1	ATCAGAGGCTGCTCGTGTTGTA	22	50	61.73
nCoV-2019_7_LEFT_alt0	nCoV-2019_1	CATTTGCATCAGAGGCTGCTCG	22	54.55	62.44
nCoV-2019_7_RIGHT	nCoV-2019_1	TGCACAGGTGACAATTTGTCCA	22	45.45	60.95
nCoV-2019_7_RIGHT_alt5	nCoV-2019_1	AGGTGACAATTTGTCCACCGAC	22	50	61.07
nCoV-2019_8_LEFT	nCoV-2019_2	AGAGTTTCTTAGAGACGGTTGGGA	24	45.83	61
nCoV-2019_8_RIGHT	nCoV-2019_2	GCTTCAACAGCTTCACTAGTAGGT	24	45.83	60.56
nCoV-2019_9_LEFT	nCoV-2019_1	TCCCACAGAAGTGTTAACAGAGGA	24	45.83	61.18
nCoV-2019_9_LEFT_alt4	nCoV-2019_1	TTCCCACAGAAGTGTTAACAGAGG	24	45.83	60.44
nCoV-2019_9_RIGHT	nCoV-2019_1	ATGACAGCATCTGCCACAACAC	22	50	61.71
nCoV-2019_9_RIGHT_alt2	nCoV-2019_1	GACAGCATCTGCCACAACACAG	22	54.55	62.26
nCoV-2019_10_LEFT	nCoV-2019_2	TGAGAAGTGCTCTGCCTATACAGT	24	45.83	61.12
nCoV-2019_10_RIGHT	nCoV-2019_2	TCATCTAACCAATCTTCTTCTTGCTCT	27	37.04	60.31
nCoV-2019_11_LEFT	nCoV-2019_1	GGAATTTGGTGCCACTTCTGCT	22	50	61.66
nCoV-2019_11_RIGHT	nCoV-2019_1	TCATCAGATTCAACTTGCATGGCA	24	41.67	61.35
nCoV-2019_12_LEFT	nCoV-2019_2	AAACATGGAGGAGGTGTTGCAG	22	50	61.08
nCoV-2019_12_RIGHT	nCoV-2019_2	TTCACTCTTCATTTCCAAAAAGCTTGA	27	33.33	60.36
nCoV-2019_13_LEFT	nCoV-2019_1	TCGCACAAATGTCTACTTAGCTGT	24	41.67	60.56
nCoV-2019_13_RIGHT	nCoV-2019_1	ACCACAGCAGTTAAAACACCCT	22	45.45	60.36
nCoV-2019_14_LEFT	nCoV-2019_2	CATCCAGATTCTGCCACTCTTGT	23	47.83	60.62

nCoV-2019_14_LEFT_alt4	nCoV-2019_2	TGGCAATCTTCATCCAGATTCTGC	24	45.83	61.47
nCoV-2019_14_RIGHT	nCoV-2019_2	AGTTTCCACACAGACAGGCATT	22	45.45	60.42
nCoV-2019_14_RIGHT_alt2	nCoV-2019_2	TGCGTGTTTCTTCTGCATGTGC	22	50	62.76
nCoV-2019_15_LEFT	nCoV-2019_1	ACAGTGCTTAAAAAGTGTAAAAGTGCC	27	37.04	61.32
nCoV-2019_15_LEFT_alt1	nCoV-2019_1	AGTGCTTAAAAAGTGTAAAAGTGCCT	26	34.62	60.13
nCoV-2019_15_RIGHT	nCoV-2019_1	AACAGAAACTGTAGCTGGCACT	22	45.45	60.16
nCoV-2019_15_RIGHT_alt3	nCoV-2019_1	ACTGTAGCTGGCACTTTGAGAGA	23	47.83	61.57
nCoV-2019_16_LEFT	nCoV-2019_2	AATTTGGAAGAAGCTGCTCGGT	22	45.45	60.82
nCoV-2019_16_RIGHT	nCoV-2019_2	CACAACTTGCGTGTGGAGGTTA	22	50	61.32
nCoV-2019_17_LEFT	nCoV-2019_1	CTTCTTTCTTTGAGAGAAGTGAGGACT	27	40.74	60.69
nCoV-2019_17_RIGHT	nCoV-2019_1	TTTGTTGGAGTGTTAACAATGCAGT	25	36	60.11
nCoV-2019_18_LEFT	nCoV-2019_2	TGGAAATACCCACAAGTTAATGGTTTAAC	29	34.48	60.69
nCoV-2019_18_LEFT_alt2	nCoV-2019_2	ACTTCTATTAAATGGGCAGATAACAACTGT	30	33.33	61.38
nCoV-2019_18_RIGHT	nCoV-2019_2	AGCTTGTTTACCACACGTACAAGG	24	45.83	61.51
nCoV-2019_18_RIGHT_alt1	nCoV-2019_2	GCTTGTTTACCACACGTACAAGG	23	47.83	60.3
nCoV-2019_19_LEFT	nCoV-2019_1	GCTGTTATGTACATGGGCACACT	23	47.83	61.18
nCoV-2019_19_RIGHT	nCoV-2019_1	TGTCCAACTTAGGGTCAATTTCTGT	25	40	60.4
nCoV-2019_20_LEFT	nCoV-2019_2	ACAAAGAAAACAGTTACACAACAACCA	27	33.33	60.68
nCoV-2019_20_RIGHT	nCoV-2019_2	ACGTGGCTTTATTAGTTGCATTGTT	25	36	60.28
nCoV-2019_21_LEFT	nCoV-2019_1	TGGCTATTGATTATAAACACTACACACCC	29	37.93	61.49
nCoV-2019_21_LEFT_alt2	nCoV-2019_1	GGCTATTGATTATAAACACTACACACCCT	29	37.93	61.29
nCoV-2019_21_RIGHT	nCoV-2019_1	TAGATCTGTGTGGCCAACCTCT	22	50	60.83
nCoV-2019_21_RIGHT_alt0	nCoV-2019_1	GATCTGTGTGGCCAACCTCTTC	22	54.55	61.2
nCoV-2019_22_LEFT	nCoV-2019_2	ACTACCGAAGTTGTAGGAGACATTATACT	29	37.93	61.25
nCoV-2019_22_RIGHT	nCoV-2019_2	ACAGTATTCTTTGCTATAGTAGTCGGC	27	40.74	60.73
nCoV-2019_23_LEFT	nCoV-2019_1	ACAACTACTAACATAGTTACACGGTGT	27	37.04	60.26
nCoV-2019_23_RIGHT	nCoV-2019_1	ACCAGTACAGTAGGTTGCAATAGTG	25	44	60.57
nCoV-2019_24_LEFT	nCoV-2019_2	AGGCATGCCTTCTTACTGTACTG	23	47.83	60.37
nCoV-2019_24_RIGHT	nCoV-2019_2	ACATTCTAACCATAGCTGAAATCGGG	26	42.31	61.19
nCoV-2019_25_LEFT	nCoV-2019_1	GCAATTGTTTTTCAGCTATTTTGCAGT	27	33.33	60.73
nCoV-2019_25_RIGHT	nCoV-2019_1	ACTGTAGTGACAAGTCTCTCGCA	23	47.83	61.3
nCoV-2019_26_LEFT	nCoV-2019_2	TTGTGATACATTCTGTGCTGGTAGT	25	40	60.28

nCoV-2019_26_RIGHT	nCoV-2019_2	TCCGCACTATCACCAACATCAG	22	50	60.42
nCoV-2019_27_LEFT	nCoV-2019_1	ACTACAGTCAGCTTATGTGTCAACC	25	44	60.8
nCoV-2019_27_RIGHT	nCoV-2019_1	AATACAAGCACCAAGGTCACGG	22	50	61.13
nCoV-2019_28_LEFT	nCoV-2019_2	ACATAGAAGTTACTGGCGATAGTTGT	26	38.46	60.13
nCoV-2019_28_RIGHT	nCoV-2019_2	TGTTTAGACATGACATGAACAGGTGT	26	38.46	60.91
nCoV-2019_29_LEFT	nCoV-2019_1	ACTTGTGTTCCTTTTTGTTGCTGC	24	41.67	61.39
nCoV-2019_29_RIGHT	nCoV-2019_1	AGTGTACTCTATAAGTTTTGATGGTGTGT	29	34.48	60.69
nCoV-2019_30_LEFT	nCoV-2019_2	GCACAACTAATGGTGACTTTTTGCA	25	40	61.19
nCoV-2019_30_RIGHT	nCoV-2019_2	ACCACTAGTAGATACACAAACACCAG	26	42.31	60.3
nCoV-2019_31_LEFT	nCoV-2019_1	TTCTGAGTACTGTAGGCACGGC	22	54.55	62.03
nCoV-2019_31_RIGHT	nCoV-2019_1	ACAGAATAAACACCAGGTAAGAATGAGT	28	35.71	60.69
nCoV-2019_32_LEFT	nCoV-2019_2	TGGTGAATACAGTCATGTAGTTGCC	25	44	61.09
nCoV-2019_32_RIGHT	nCoV-2019_2	AGCACATCACTACGCAACTTTAGA	24	41.67	60.56
nCoV-2019_33_LEFT	nCoV-2019_1	ACTTTTGAAGAAGCTGCGCTGT	22	45.45	61.58
nCoV-2019_33_RIGHT	nCoV-2019_1	TGGACAGTAAACTACGTCATCAAGC	25	44	61.08
nCoV-2019_34_LEFT	nCoV-2019_2	TCCCATCTGGTAAAGTTGAGGGT	23	47.83	61.02
nCoV-2019_34_RIGHT	nCoV-2019_2	AGTGAAATTGGGCCTCATAGCA	22	45.45	60.03
nCoV-2019_35_LEFT	nCoV-2019_1	TGTTCGCATTCAACCAGGACAG	22	50	61.39
nCoV-2019_35_RIGHT	nCoV-2019_1	ACTTCATAGCCACAAGGTTAAAGTCA	26	38.46	60.69
nCoV-2019_36_LEFT	nCoV-2019_2	TTAGCTTGGTTGTACGCTGCTG	22	50	61.44
nCoV-2019_36_RIGHT	nCoV-2019_2	GAACAAAGACCATTGAGTACTCTGGA	26	42.31	60.74
nCoV-2019_37_LEFT	nCoV-2019_1	ACACACCACTGGTTGTTACTCAC	23	47.83	60.93
nCoV-2019_37_RIGHT	nCoV-2019_1	GTCCACACTCTCCTAGCACCAT	22	54.55	61.48
nCoV-2019_38_LEFT	nCoV-2019_2	ACTGTGTTATGTATGCATCAGCTGT	25	40	60.86
nCoV-2019_38_RIGHT	nCoV-2019_2	CACCAAGAGTCAGTCTAAAGTAGCG	25	48	61.13
nCoV-2019_39_LEFT	nCoV-2019_1	AGTATTGCCCTATTTTCTTCATAACTGGT	29	34.48	61
nCoV-2019_39_RIGHT	nCoV-2019_1	TGTAACTGGACACATTGAGCCC	22	50	60.55
nCoV-2019_40_LEFT	nCoV-2019_2	TGCACATCAGTAGTCTTACTCTCAGT	26	42.31	61.25
nCoV-2019_40_RIGHT	nCoV-2019_2	CATGGCTGCATCACGGTCAAAT	22	50	62.09
nCoV-2019_41_LEFT	nCoV-2019_1	GTTCCCTTCCATCATATGCAGCT	23	47.83	60.75
nCoV-2019_41_RIGHT	nCoV-2019_1	TGGTATGACAACCATTAGTTTGGCT	25	40	60.75
nCoV-2019_42_LEFT	nCoV-2019_2	TGCAAGAGATGGTTGTGTTCCC	22	50	61.08

nCoV-2019_42_RIGHT	nCoV-2019_2	CCTACCTCCCTTTGTTGTGTTGT	23	47.83	60.69
nCoV-2019_43_LEFT	nCoV-2019_1	TACGACAGATGTCTTGTGCTGC	22	50	60.93
nCoV-2019_43_RIGHT	nCoV-2019_1	AGCAGCATCTACAGCAAAAGCA	22	45.45	61.14
nCoV-2019_44_LEFT	nCoV-2019_2	TGCCACAGTACGTCTACAAGCT	22	50	61.66
nCoV-2019_44_LEFT_alt3	nCoV-2019_2	CCACAGTACGTCTACAAGCTGG	22	54.55	60.67
nCoV-2019_44_RIGHT	nCoV-2019_2	AACCTTTCCACATACCGCAGAC	22	50	60.87
nCoV-2019_44_RIGHT_alt0	nCoV-2019_2	CGCAGACGGTACAGACTGTGTT	22	54.55	62.77
nCoV-2019_45_LEFT	nCoV-2019_1	TACCTACAACTTGTGCTAATGACCC	25	44	60.57
nCoV-2019_45_LEFT_alt2	nCoV-2019_1	AGTATGTACAAATACCTACAACTTGTGCT	29	34.48	60.94
nCoV-2019_45_RIGHT	nCoV-2019_1	AAATTGTTTCTTCATGTTGGTAGTTAGAGA	30	30	60.01
nCoV-2019_45_RIGHT_alt7	nCoV-2019_1	TTCATGTTGGTAGTTAGAGAAAGTGTGTC	29	37.93	61.53
nCoV-2019_46_LEFT	nCoV-2019_2	TGTCGCTTCCAAGAAAAGGACG	22	50	61.38
nCoV-2019_46_LEFT_alt1	nCoV-2019_2	CGCTTCCAAGAAAAGGACGAAGA	23	47.83	61.35
nCoV-2019_46_RIGHT	nCoV-2019_2	CACGTTCACCTAAGTTGGCGTA	22	50	60.86
nCoV-2019_46_RIGHT_alt2	nCoV-2019_2	CACGTTCACCTAAGTTGGCGTAT	23	47.83	61.17
nCoV-2019_47_LEFT	nCoV-2019_1	AGGACTGGTATGATTTTGTAGAAAACCC	28	39.29	61.42
nCoV-2019_47_RIGHT	nCoV-2019_1	AATAACGGTCAAAGAGTTTTAACCTCTC	28	35.71	60.06
nCoV-2019_48_LEFT	nCoV-2019_2	TGTTGACACTGACTTAACAAAGCCT	25	40	61.09
nCoV-2019_48_RIGHT	nCoV-2019_2	TAGATTACCAGAAGCAGCGTGC	22	50	60.74
nCoV-2019_49_LEFT	nCoV-2019_1	AGGAATTACTTGTGTATGCTGCTGA	25	40	60.57
nCoV-2019_49_RIGHT	nCoV-2019_1	TGACGATGACTTGGTTAGCATTAATACA	28	35.71	61.05
nCoV-2019_50_LEFT	nCoV-2019_2	GTTGATAAGTACTTTGATTGTTACGATGGT	30	33.33	60.59
nCoV-2019_50_RIGHT	nCoV-2019_2	TAACATGTTGTGCCAACCACCA	22	45.45	60.95
nCoV-2019_51_LEFT	nCoV-2019_1	TCAATAGCCGCCACTAGAGGAG	22	54.55	61.34
nCoV-2019_51_RIGHT	nCoV-2019_1	AGTGCATTAACATTGGCCGTGA	22	45.45	61.14
nCoV-2019_52_LEFT	nCoV-2019_2	CATCAGGAGATGCCACAACTGC	22	54.55	61.83
nCoV-2019_52_RIGHT	nCoV-2019_2	GTTGAGAGCAAAATTCATGAGGTCC	25	44	60.62
nCoV-2019_53_LEFT	nCoV-2019_1	AGCAAAATGTTGGACTGAGACTGA	24	41.67	60.69
nCoV-2019_53_RIGHT	nCoV-2019_1	AGCCTCATAAAACTCAGGTTCCC	23	47.83	60.31
nCoV-2019_54_LEFT	nCoV-2019_2	TGAGTTAACAGGACACATGTTAGACA	26	38.46	60.18
nCoV-2019_54_RIGHT	nCoV-2019_2	AACCAAAAACTTGTCCATTAGCACA	25	36	60.11
nCoV-2019_55_LEFT	nCoV-2019_1	ACTCAACTTTACTTAGGAGGTATGAGCT	28	39.29	61.43

nCoV-2019_55_RIGHT	nCoV-2019_1	GGTGTACTCTCCTATTTGTACTTTACTGT	29	37.93	60.54
nCoV-2019_56_LEFT	nCoV-2019_2	ACCTAGACCACCACTTAACCGA	22	50	60.49
nCoV-2019_56_RIGHT	nCoV-2019_2	ACACTATGCGAGCAGAAGGGTA	22	50	61.21
nCoV-2019_57_LEFT	nCoV-2019_1	ATTCTACACTCCAGGGACCACC	22	54.55	61.16
nCoV-2019_57_RIGHT	nCoV-2019_1	GTAATTGAGCAGGGTCGCCAAT	22	50	61.26
nCoV-2019_58_LEFT	nCoV-2019_2	TGATTTGAGTGTTGTCAATGCCAGA	25	40	61.44
nCoV-2019_58_RIGHT	nCoV-2019_2	CTTTTCTCCAAGCAGGGTTACGT	23	47.83	61.06
nCoV-2019_59_LEFT	nCoV-2019_1	TCACGCATGATGTTTCATCTGCA	23	43.48	61.42
nCoV-2019_59_RIGHT	nCoV-2019_1	AAGAGTCCTGTTACATTTTCAGCTTG	26	38.46	60.02
nCoV-2019_60_LEFT	nCoV-2019_2	TGATAGAGACCTTTATGACAAGTTGCA	27	37.04	60.53
nCoV-2019_60_RIGHT	nCoV-2019_2	GGTACCAACAGCTTCTCTAGTAGC	24	50	60.44
nCoV-2019_61_LEFT	nCoV-2019_1	TGTTTATCACCCGCGAAGAAGC	22	50	61.5
nCoV-2019_61_RIGHT	nCoV-2019_1	ATCACATAGACAACAGGTGCGC	22	50	61.25
nCoV-2019_62_LEFT	nCoV-2019_2	GGCACATGGCTTTGAGTTGACA	22	50	61.91
nCoV-2019_62_RIGHT	nCoV-2019_2	GTTGAACCTTTCTACAAGCCGC	22	50	60.35
nCoV-2019_63_LEFT	nCoV-2019_1	TGTTAAGCGTGTTGACTGGACT	22	45.45	60.16
nCoV-2019_63_RIGHT	nCoV-2019_1	ACAAACTGCCACCATCACAACC	22	50	61.85
nCoV-2019_64_LEFT	nCoV-2019_2	TCGATAGATATCCTGCTAATTCCATTGT	28	35.71	60.11
nCoV-2019_64_RIGHT	nCoV-2019_2	AGTCTTGTAAAAGTGTTCCAGAGGT	25	40	60.1
nCoV-2019_65_LEFT	nCoV-2019_1	GCTGGCTTTAGCTTGTGGGTTT	22	50	61.92
nCoV-2019_65_RIGHT	nCoV-2019_1	TGTCAGTCATAGAACAAACACCAATAGT	28	35.71	60.9
nCoV-2019_66_LEFT	nCoV-2019_2	GGGTGTGGACATTGCTGCTAAT	22	50	61.21
nCoV-2019_66_RIGHT	nCoV-2019_2	TCAATTTCCATTTGACTCCTGGGT	24	41.67	60.45
nCoV-2019_67_LEFT	nCoV-2019_1	GTTGTCCAACAATTACCTGAAACTTACT	28	35.71	60.43
nCoV-2019_67_RIGHT	nCoV-2019_1	CAACCTTAGAAACTACAGATAAATCTTGGG	30	36.67	60.4
nCoV-2019_68_LEFT	nCoV-2019_2	ACAGGTTCATCTAAGTGTGTGTGT	24	41.67	60.14
nCoV-2019_68_RIGHT	nCoV-2019_2	CTCCTTTATCAGAACCAGCACCA	23	47.83	60.31
nCoV-2019_69_LEFT	nCoV-2019_1	TGTCGCAAAATATACTCAACTGTGTCA	27	37.04	61.43
nCoV-2019_69_RIGHT	nCoV-2019_1	TCTTTATAGCCACGGAACCTCCA	23	47.83	61.14
nCoV-2019_70_LEFT	nCoV-2019_2	ACAAAAGAAAATGACTCTAAAGAGGGTTT	29	31.03	60.13
nCoV-2019_70_RIGHT	nCoV-2019_2	TGACCTTCTTTTAAAGACATAACAGCAG	28	35.71	60.27
nCoV-2019_71_LEFT	nCoV-2019_1	ACAAATCCAATTCAGTTGTCTTCCTATTC	29	34.48	60.54

nCoV-2019_71_RIGHT	nCoV-2019_1	TGGAAAAGAAAGGTAAGAACAAGTCCT	27	37.04	60.8
nCoV-2019_72_LEFT	nCoV-2019_2	ACACGTGGTGTTTATTACCCTGAC	24	45.83	61.04
nCoV-2019_72_RIGHT	nCoV-2019_2	ACTCTGAACTCACTTTCCATCCAAC	25	44	60.97
nCoV-2019_73_LEFT	nCoV-2019_1	CAATTTTGTAATGATCCATTTTTGGGTGT	29	31.03	60.29
nCoV-2019_73_RIGHT	nCoV-2019_1	CACCAGCTGTCCAACCTGAAGA	22	54.55	62.45
nCoV-2019_74_LEFT	nCoV-2019_2	ACATCACTAGGTTTCAAACTTTACTTGC	28	35.71	60.68
nCoV-2019_74_RIGHT	nCoV-2019_2	GCAACACAGTTGCTGATTCTCTTC	24	45.83	60.85
nCoV-2019_75_LEFT	nCoV-2019_1	AGAGTCCAACCAACAGAATCTATTGT	26	38.46	60.24
nCoV-2019_75_RIGHT	nCoV-2019_1	ACCACCAACCTTAGAATCAAGATTGT	26	38.46	60.69
nCoV-2019_76_LEFT	nCoV-2019_2	AGGGCAAACTGGAAAGATTGCT	22	45.45	60.76
nCoV-2019_76_LEFT_alt3	nCoV-2019_2	GGGCAAACTGGAAAGATTGCTGA	23	47.83	61.87
nCoV-2019_76_RIGHT	nCoV-2019_2	ACACCTGTGCCTGTTAAACCAT	22	45.45	60.42
nCoV-2019_76_RIGHT_alt0	nCoV-2019_2	ACCTGTGCCTGTTAAACCATTGA	23	43.48	60.69
nCoV-2019_77_LEFT	nCoV-2019_1	CCAGCAACTGTTTGTGGACCTA	22	50	60.75
nCoV-2019_77_RIGHT	nCoV-2019_1	CAGCCCCTATTAAACAGCCTGC	22	54.55	61.59
nCoV-2019_78_LEFT	nCoV-2019_2	CAACTTACTCCTACTTGGCGTGT	23	47.83	60.55
nCoV-2019_78_RIGHT	nCoV-2019_2	TGTGTACAAAAACTGCCATATTGCA	25	36	60.22
nCoV-2019_79_LEFT	nCoV-2019_1	GTGGTGATTCAACTGAATGCAGC	23	47.83	60.92
nCoV-2019_79_RIGHT	nCoV-2019_1	CATTTCATCTGTGAGCAAAGGTGG	24	45.83	60.62
nCoV-2019_80_LEFT	nCoV-2019_2	TTGCCTTGGTGATATTGCTGCT	22	45.45	60.89
nCoV-2019_80_RIGHT	nCoV-2019_2	TGGAGCTAAGTTGTTTAACAAGCG	24	41.67	60.02
nCoV-2019_81_LEFT	nCoV-2019_1	GCACTTGGAAAACTTCAAGATGTGG	25	44	61.24
nCoV-2019_81_RIGHT	nCoV-2019_1	GTGAAGTTCTTTTCTTGTGCAGGG	24	45.83	60.73
nCoV-2019_82_LEFT	nCoV-2019_2	GGGCTATCATCTTATGTCCTTCCCT	25	48	61.52
nCoV-2019_82_RIGHT	nCoV-2019_2	TGCCAGAGATGTCACCTAAATCAA	24	41.67	60.02
nCoV-2019_83_LEFT	nCoV-2019_1	TCCTTTGCAACCTGAATTAGACTCA	25	40	60.46
nCoV-2019_83_RIGHT	nCoV-2019_1	TTTGACTCCTTTGAGCACTGGC	22	50	61.33
nCoV-2019_84_LEFT	nCoV-2019_2	TGCTGTAGTTGTCTCAAGGGCT	22	50	61.61
nCoV-2019_84_RIGHT	nCoV-2019_2	AGGTGTGAGTAAACTGTTACAAACAAC	27	37.04	60.36
nCoV-2019_85_LEFT	nCoV-2019_1	ACTAGCACTCTCCAAGGGTGTT	22	50	61.03
nCoV-2019_85_RIGHT	nCoV-2019_1	ACACAGTCTTTTACTCCAGATTCCC	25	44	60.51
nCoV-2019_86_LEFT	nCoV-2019_2	TCAGGTGATGGCACAACAAGTC	22	50	61.07

nCoV-2019_86_RIGHT	nCoV-2019_2	ACGAAAGCAAGAAAAAGAAGTACGC	25	40	61.01
nCoV-2019_87_LEFT	nCoV-2019_1	CGACTACTAGCGTGCCTTTGTA	22	50	60.16
nCoV-2019_87_RIGHT	nCoV-2019_1	ACTAGGTTCCATTGTTCAAGGAGC	24	45.83	60.81
nCoV-2019_88_LEFT	nCoV-2019_2	CCATGGCAGATTCCAACGGTAC	22	54.55	61.58
nCoV-2019_88_RIGHT	nCoV-2019_2	TGGTCAGAATAGTGCCATGGAGT	23	47.83	61.4
nCoV-2019_89_LEFT	nCoV-2019_1	GTACGCGTTCCATGTGGTCATT	22	50	61.5
nCoV-2019_89_LEFT_alt2	nCoV-2019_1	CGCGTTCCATGTGGTCATTCAA	22	50	62.01
nCoV-2019_89_RIGHT	nCoV-2019_1	ACCTGAAAGTCAACGAGATGAAACA	25	40	60.91
nCoV-2019_89_RIGHT_alt4	nCoV-2019_1	ACGAGATGAAACATCTGTTGTCACT	25	40	60.74
nCoV-2019_90_LEFT	nCoV-2019_2	ACACAGACCATTCCAGTAGCAGT	23	47.83	61.58
nCoV-2019_90_RIGHT	nCoV-2019_2	TGAAATGGTGAATTGCCCTCGT	22	45.45	60.82
nCoV-2019_91_LEFT	nCoV-2019_1	TCACTACCAAGAGTGTGTTAGAGGT	25	44	60.93
nCoV-2019_91_RIGHT	nCoV-2019_1	TTCAAGTGAGAACCAAAAGATAATAAGCA	29	31.03	60.03
nCoV-2019_92_LEFT	nCoV-2019_2	TTTGTGCTTTTTAGCCTTTCTGCT	24	37.5	60.14
nCoV-2019_92_RIGHT	nCoV-2019_2	AGGTTCCTGGCAATTAATTGTAAAAGG	27	37.04	60.53
nCoV-2019_93_LEFT	nCoV-2019_1	TGAGGCTGGTTCTAAATCACCCA	23	47.83	61.59
nCoV-2019_93_RIGHT	nCoV-2019_1	AGGTCTTCCTTGCCATGTTGAG	22	50	60.55
nCoV-2019_94_LEFT	nCoV-2019_2	GGCCCCAAGGTTTACCCAATAA	22	50	60.56
nCoV-2019_94_RIGHT	nCoV-2019_2	TTTGGCAATGTTGTTCCTTGAGG	23	43.48	60.18
nCoV-2019_95_LEFT	nCoV-2019_1	TGAGGGAGCCTTGAATACACCA	22	50	61.1
nCoV-2019_95_RIGHT	nCoV-2019_1	CAGTACGTTTTTGCCGAGGCTT	22	50	61.95
nCoV-2019_96_LEFT	nCoV-2019_2	GCCAACAACAACAAGGCCAAAC	22	50	61.82
nCoV-2019_96_RIGHT	nCoV-2019_2	TAGGCTCTGTTGGTGGGAATGT	22	50	61.36
nCoV-2019_97_LEFT	nCoV-2019_1	TGGATGACAAAGATCCAAATTTCAAAGA	28	32.14	60.22
nCoV-2019_97_RIGHT	nCoV-2019_1	ACACACTGATTAAAGATTGCTATGTGAG	28	35.71	60.17
nCoV-2019_98_LEFT	nCoV-2019_2	AACAATTGCAACAATCCATGAGCA	24	37.5	60.5
nCoV-2019_98_RIGHT	nCoV-2019_2	TTCTCCTAAGAAGCTATTAAAATCACATGG	30	33.33	60.01

Supplementary Table 3. ARTIC v3 primer sequences.
